# Curing of DER-331 Epoxy Resin with Arylaminocyclotriphosphazenes Based on o-, m-, and p-methylanilines

**DOI:** 10.3390/polym14245334

**Published:** 2022-12-07

**Authors:** Artem A. Rybyan, Julia V. Bilichenko, Vyacheslav V. Kireev, Alexander A. Kolenchenko, Evgeniy M. Chistyakov

**Affiliations:** Faculty of Petrochemistry and Polymer Materials, Mendeleev University of Chemical Technology, 125047 Moscow, Russia

**Keywords:** cyclotriphosphazenes, aminocyclotriphosphazenes, deaminolysis, epoxy resin, modifiers

## Abstract

As a result of this research, it was established that the chlorine atom replacement rates in hexa-chlorocyclotriphosphazene by o-, m-, and p-methylanilines’ temperatures are crucial in determining which reaction is made. The speed of reaction practically does not affect the polarity of the synthesis solvent. For the formation of fully substituted o-, m-, and p-arilaminocyclotriphosphazenes, the reaction takes 5 h and is carried out in the diglyme at its boiling temperature. The structure of the synthesized AAP was confirmed by ^31^P and ^1^H NMR spectroscopy and MALDI-TOF mass spectrometry. By means of synchronous DSK and TGA, it is found that the synthesized AAP are crystalline and their thermal destruction has a stepped character. Thermal destruction is shown to be accompanied by the simultaneous removal of three aniline molecules from the AAP molecules. Conducted curing of epoxy resin DER-331 is carried out using the AAP as a curing agent. It has been established that due to steric difficulties, o- AAP does not interact with epoxy resin, unlike m- and p- AAP. The gel fraction in curing resin is measured, and the AAP relate to the stage processes of macromolecule formation. The result is that polymers based on DER-331 and m-, p-AAP have a gel fraction content up to 97 mass. %. These polymers have glass-transition temperatures 80 and 85 °C (m- and p-AAP-based, respectively) and demonstrate fire resistance to standard UL-94 of category V-0.

## 1. Introduction

Modern polymer materials are increasingly in demand in many fields of science, technology, and medicine. Examples of such materials and studies include photosensitive supramolecular hydrogels [1], research reokinetics [2], the synthesis of effective substrates for the oxidation of thiophenols and benzylthioles before their respective disulphides in oxygen air [3], the development of new recovery systems for oil and gas resources [4], research mixture epoxy diluents with the benzoxazine monomer on the obtained by curing matrix [5], the development of a method for the production of metal foams [6], the investigation of the mechanical characteristics of polymer stents with blood-flow dynamics [7], and the effectiveness evaluation of injection hydrogel [8]. Among thermoset polymer materials, the most interesting are epoxy resins and composites based on them through simple recycling, the opportunity to change the properties’ composite by combining epoxy resin and the curing agent, and their good operational characteristics [9,10,11]. For example, bio-based epoxy resin, which is 34% biocomponents, mixed with conventional epoxy resin, has high hardness, as well as tensile and bending strength [12]. Epoxy-coatings-modified nanoparticles aluminum have great corrosion resistance even after 10 days in salt water and after exposure to icing\de-icing process [13]. In a resin containing 10% aluminum hydroxide and an ammonium polyphosphate mass ratio of 3/1, peak heat generation and total heat generation decreased by 48% and 38%. This opens up the prospect of using such compositions for electronic devices [14]. Epoxy compounds provide stable material with free epoxy groups, which can serve as a carrier of recombinant protein-growth factors under conditions of pronounced regenerative process [15]. Polymers and composites produced by the cyclopolymerization of epoxy resins with different di- and polyisocyanates have high heat resistant due to the presence of cyclic oxazolidines and isocyanurates in their molecular structure [16]. However, cured epoxy resins do not always have a sufficient glass transition temperature for use in various areas of technology, which necessitates their modification. Organo-substituted cyclotrophosphazenes are of continuing interest in chemical science because they can be used as effective modifiers for various composites and polymer materials [17]. Aminocyclotriphosphazenes capable of providing low-fuel and low-glass-transition-temperature compositions are a promising class of organophosphazenes [18]. HCP-based compositions can be produced with a range of fuel-free compositions: high thermal, chemical, corrosion resistance, high tensile strength, adhesion, and low solidification [19]. As a result, such compounds can be used in many advanced fields of science and technology: aviation, aerospace, radioengineering, chemical, nuclear, and medical [20]. Therefore, the aim of the work was the synthesis of amino acidic rifosphazines and the testing of them as modifiers–flame retardants of industrial epoxy resins.

## 2. Materials and Methods

Hexachlorocyclotriphosphazine, 99% (Fushimi Pharmaceutical Co., Ltd., Maru Game, Kagawa Prefecture, Japan); diethylene glycol dimethyl ether (diglyme), 99%; 2-methylaniline, 99%; 3- methylaniline, 99%; 4- methylaniline, 99%; triethylamine, 99%; and epoxy resin DER-331, EEW = 170.63 gE, petroleum ether bp ≥ 90% 40–60 °C (≥90%), and methylene chloride, 99% (Sigma-Aldrich, Saint Louis, MO, USA).

In total, 10 g (0.0287 mol) HCP, 24.57 mL methylaniline (0.2296 mol), and 24.4 mL triethylamine (0.1751 mol) were added to a double-walled 250 mL round-bottom flask equipped with a reflux condenser and a top drive mixer and dissolved into components in 40 mL diglyme. Synthesis was carried out within 5 h at the boiling point of the solvent. The AAP were white powders. o-AAP was purified from 2-methylaniline washing ethyl alcohol. m-AAP and p-AAP washing mixture methylene chloride: petroleum ether was taken in volume ratio of 1:1. The yield o-AAP, m-AAP, and p-AAP amounted to 83%, 88%, and 89%, respectively.

^31^P and ^1^H NMR spectrometers have been detected at 600.02 MHz and 242.91 MHz (Agilent Technologies, Santa Clara, CA, USA) spectrometers.

IR spectra have been detected at the spectrometer Nicolet 380 (Thermo Fisher Scientific, Waltham, MA, USA) in a spectral range of 4000–500 cm^−1^ with an accuracy of 0.01 cm^−1^.

MALDI-TOF—mass spectra have been recorded on a reflector time-lapse mass spectrometer Bruker AutoFlex II (Bruker, Billerica, MA, USA) with ISO standard 10927:2018 in a 3-Hydroxypicolinic acid matrix.

The glass transition temperature was determined according to ISO 11357-2:2020 using a differential scanning calorimeter Netzsch DSC 204 F1 Phoenix (Erich NETZSCH GmbH and Co. Holding KG, Selb, Germany). The heating rate for all measurements was 10 deg/min. All tests were carried out within a temperature range of 30–300 °C in the nitrogen atmosphere at a flow rate of 40 mL/min.

Mass loss was determined according to ISO 7111-87 using a synchronous thermal analysis device Netzsch STA 449 F3 Jupiter (Erich NETZSCH GmbH and Co. Holding KG, Selb, Germany). The heating rate for all measurements was 10 deg/min. All tests were carried out within a temperature range of 30–800 °C in the helium atmosphere at a flow rate of 40 mL/min.

The resistance to burning of the prepared compositions was determined by the test UL-94.

The AAP pyrolysis was carried out in a capped glass ampoule filled with argon. The ampoules were held in the thermal cabinet for 15 min at a temperature of 250 °C.

The solidification of DER-331 was carried out by adding a powder corresponding to the AAP, thoroughly mixing the components and heating the resulting paste for 4 h at 200 °C.

The gel-fraction content in the cured epoxy resin was determined with the aid of the Soxlet extractor. The trial was conducted for eight hours using 1,4-dioxane as an elute.

## 3. Results and Discussion

### Reaction Conditions

**Figure 1:**
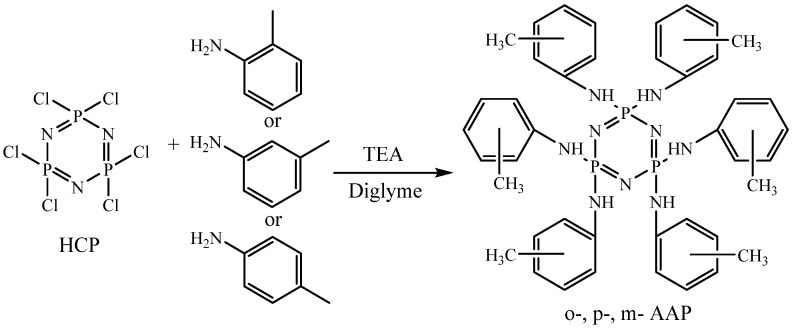
Scheme of obtaining AAP.

**Figure 2:**
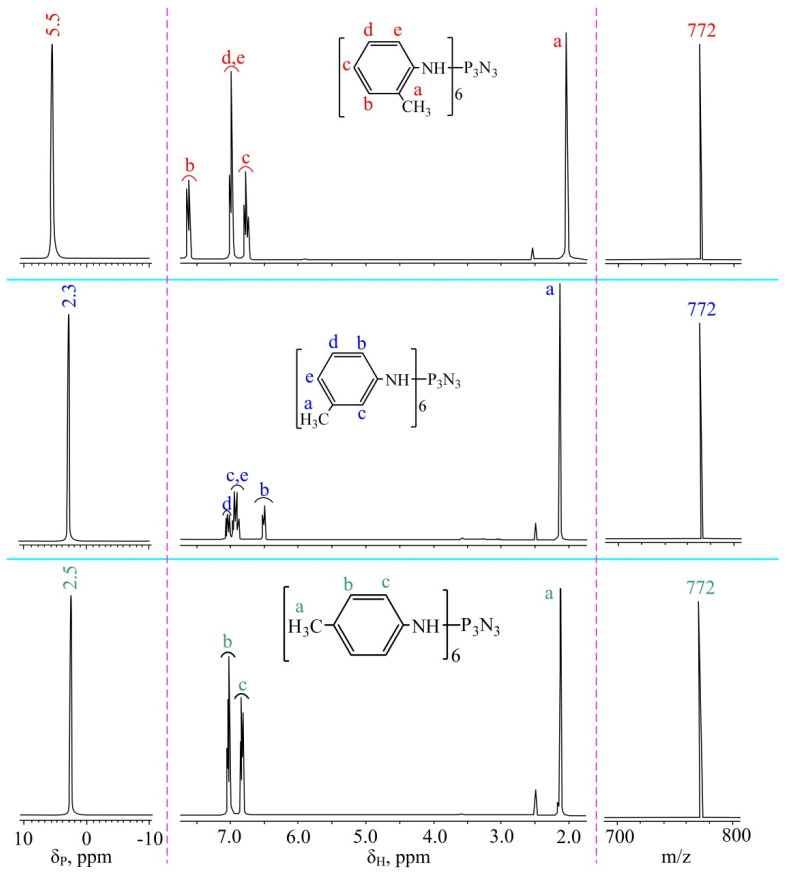
^31^P (**left**), ^1^H (in the **middle**), and MALDI-TOF (**right**) spectra o-AAP, m-AAP, and p-AAP.

The initial objective of the study was to determine the influence of solvent polarity and temperature on the reaction rate between HCP and methylanilines. This is necessary in order to find a solvent for which the substitution of chlorine atoms by methylanilines will occur most rapidly. When boiled in acetonitrile, it is known that the complete substitution of the p-methylaniline chlorine atoms in HCP takes approximately 36 h. When boiling in diethyl ether—360 h, the polarity and boiling point of acetonitrile are ε = 35.59 and T = 81 °C, respectively. For diethyl ether, these values are ε = 4.335 and T = 34 °C [21]. Since both the boiling point and the acetonitrile polarity are higher, it is difficult to conclude why the reaction is faster. Therefore, for the reaction between HCP and p-methylaniline, it was decided to use a diglyme with a polarity of ε = 5.790 and a boiling point of T = 162 °C. So, its polarity is close to diethyl ether, and its boiling point is higher than that of acetonitrile. When a reaction occurs in bis(2-methoxyethyl) ether at a boiling point of acetonitrile (81 °C), it has been established that all chlorine atoms in HCP are substituted after at least 35 h. At the boiling point of diglyme (162 °C), full chlorine substitution took only 5 h. Consequently, it can be concluded that the reaction rate between HCP and p-methylaniline is not affected by polarity but is affected by temperature. Therefore, it was decided to synthesize all of the AAP in diglyme according to the scheme presented in Figure 1. The process was monitored by ^31^P NMR spectroscopy. After 5 h of synthesis on phosphorus, the spectra of all of the AAP singlets were observed (Figure 2). The nature of the signals and their chemical shifts have not changed in the longer process.

In the case of cyclotryphosphazenes, the singlet signal on the ^31^P NMR spectrum is observed when all phosphorus atoms have the same substitutes. That is, a singlet may correspond to a non-heminal trisubstituted product rather than a hexasubstituted product. Therefore, MALDI-TOF mass spectrometry was additionally conducted. On the mass spectra of all three AAP there is a single peak with *m*/*z* = 772, corresponding to the mass of the end products (Figure 2). Consequently, the substitution of chlorine atoms in HCP for methylanilines has been completed.

To assess the presence of organic impurities and the residual solvent, ^1^H NMR spectroscopy was conducted. All three AAP spectra show only proton signals related to these compounds, confirming the purity of the substances (Figure 2).

**Figure 3:**
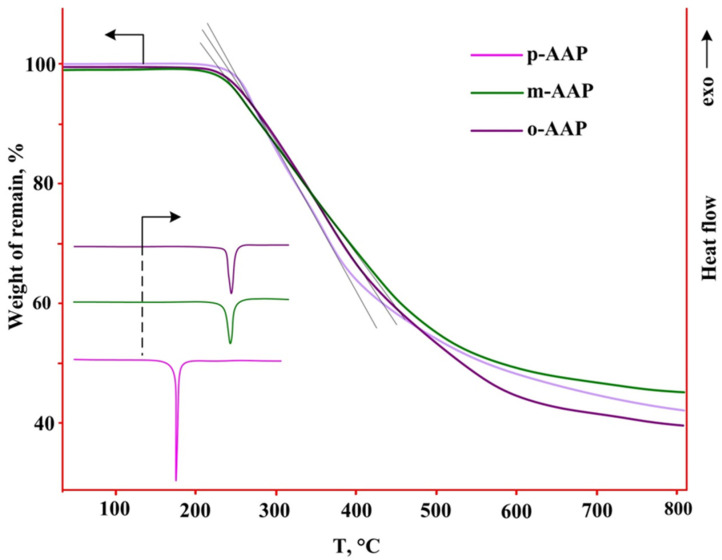
TGA and DSC curves AAP (arrows show which scale corresponds to which thermograms).

To determine the conditions for combining with epoxy resins and estimating the operating temperatures of the AAP, it was necessary to study the temperature characteristics of the phosphazenes. To this end, TGA and DSC analyses of the synthesized arilaminocyclotriphosphazenes were carried out. From TGA curves, all of the AAP are thermally stable to about 210 °C. At the same time, on the DSC thermograms it is seen that only p-AAP melts at 175 °C; for the m- and o-AAP derivatives, the melting point coincides with the decomposition starting point (Figure 3).

The TGA results also show that the thermal degradation of all of the AAP is graded, which is shown by tangent lines between curve inflection points (Figure 3). This pattern suggests the pyrolysis of the chemical process with the release of the volatile component in certain stoichiometric quantities. Since the mass loss between inflection points is in the order of 35–40%, the most likely process is to eliminate large organic fragments.

**Figure 4:**
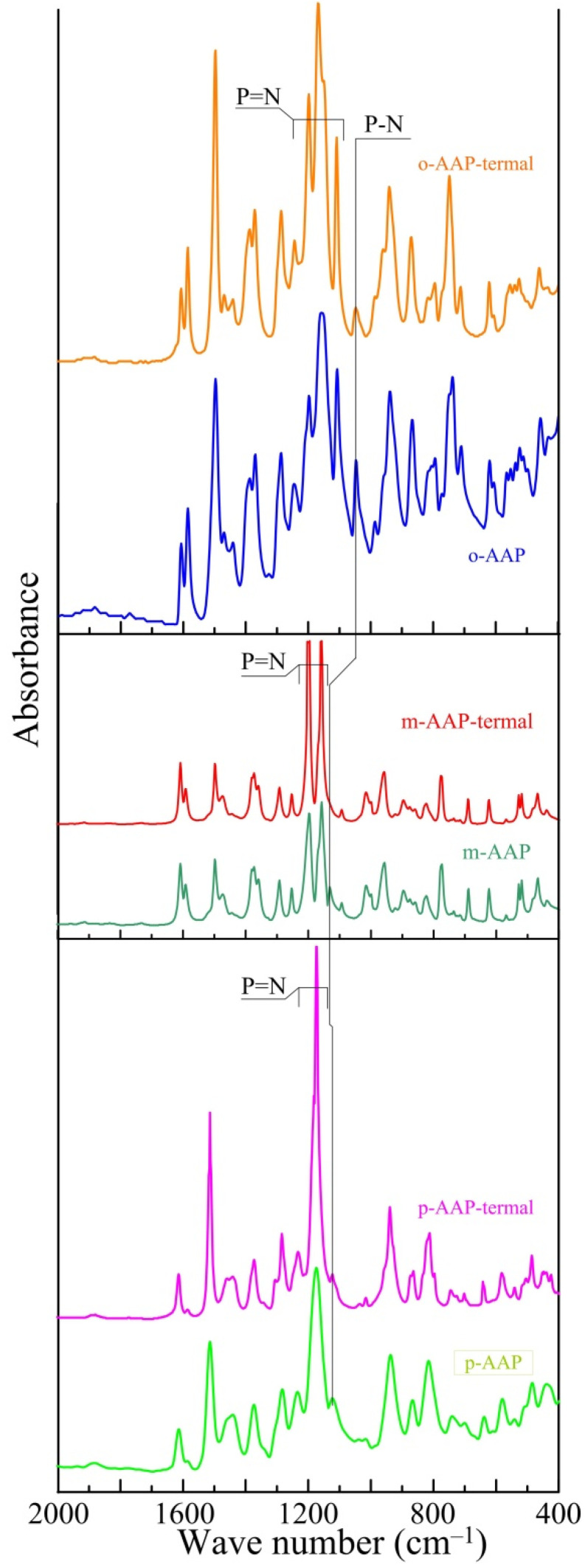
IR spectra preheating and post-heating (termal).

To determine the nature of the processes, the isothermal pyrolysis of all of the AAP was performed at a temperature of 250 °C, after which the samples were examined with IR spectroscopy (Figure 4).

**Figure 5:**
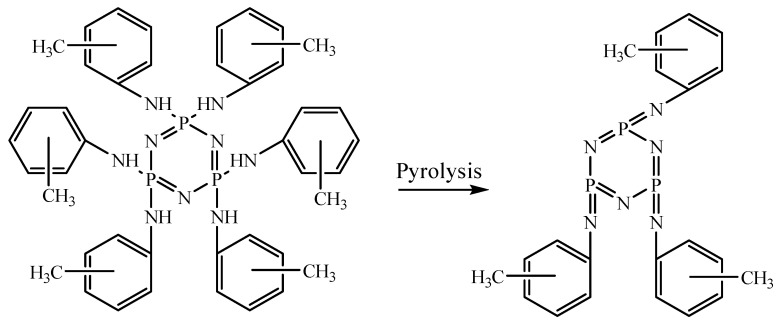
AAP pyrolysis flow circuit and the expected structure of the resulting product.

Comparing the AAP spectra before and after the warm-up, it is clear that there are no noticeable changes in the warmed-up bands. However, all three of the warmed-up AAP specimens show a decrease in the intensity of the P-N coupling bands and an increase in P=N coupling fluctuations. This may indirectly indicate a temperature-induced deaminolysis reaction forming a new phosphazene bond. Based on this loss of mass, it can be assumed that one aniline molecule is separated from each phosphorus atom, according to the scheme represented in Figure 5.

**Figure 6:**
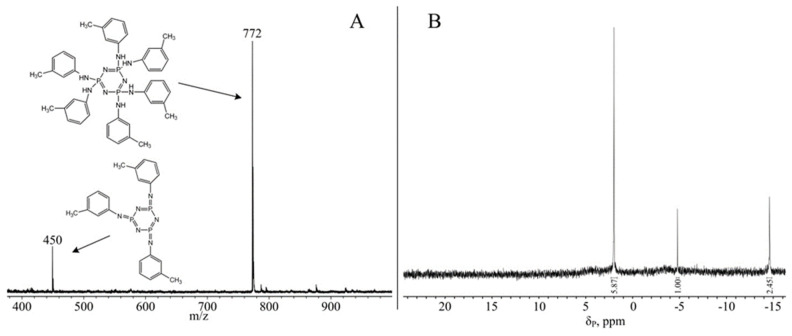
MALDI-TOF (**A**) and ^31^P NMR (**B**) spectra product pyrolysis m-AAP.

To test the hypothesis, pyrolysis products have been further investigated by 31P NMR spectroscopy and MALDI-TOF mass spectrometry. When the samples were dissolved for analysis, it was noted that the pyrolysis products were completely soluble, indicating a lack of cross-linked polymer products. By means of MALDI-TOF, it was determined that the pyrolysis at selected conditions is partially leaking as there is a signal of the initial AAP in the spectrum (Figure 6A, shown by the example m-AAP). However, there is also a peak in the spectrum corresponding to the mass of the product pyrolysis represented in Figure 5. It is noteworthy that the signals of the intermediate pyrolysis products, i.e., one or two separated aniline molecules, are absent from the spectrum.

This is also confirmed by the phosphorus spectrum, which contains a singlet corresponding to the m-AAP (Figure 6B, 2.3 ppm) phosphorus atoms as well as two other singlets that indicate the equivalence of all phosphorus atoms in the phosphazene ring, i.e., there are no intermediate pyrolysis products in the sample. Consequently, it can be concluded that when heated above 230 °C AAP, aniline molecules begin to diverge to form compounds that are potentially unsuitable for epoxy resins. This should be taken into account when choosing the curing mode.

**Figure 7:**
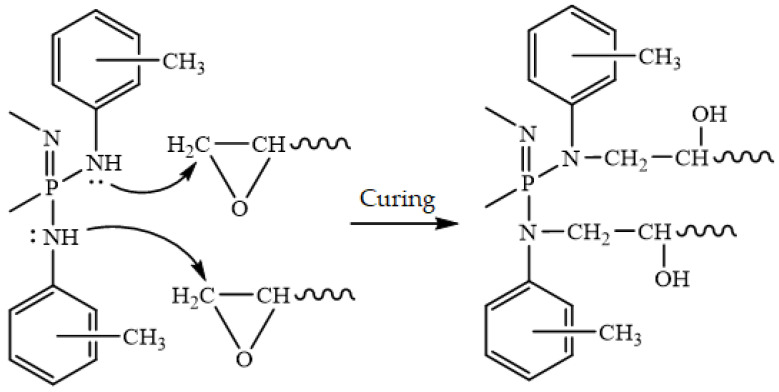
Diagram of the probable interaction of oxirane groups with AAP in the hardening of epoxy resin.

Although the nitrogen atoms of secondary amides have low nucleophilicity, they are able to react with epoxy groups. The use of ariloxysephazenes containing secondary amide groups in aromatic radicals as hardeners of epoxy resins is known [22]. Consequently, it has been suggested that -NH groups in the AAP may react with oxirane rings (Figure 7), and the AAP can also be used to solidify epoxy resins.

To test this version, it was decided to perform a solidification process using the AAP of the most common industrial resin DER-331. Since the AAP are solids, it was only possible to combine them with resin by grinding them together to form homogeneous pastes. Pastes were prepared with AAP content in resin from 5 to 15 mass. %. This is necessary in order to determine whether the curing process, as well as its mechanism, determines the gel fraction content in the cured samples. The paste was solidified at 200 °C as the AAP would dissolve completely into the resin. At higher temperatures, there was a risk of AAP decomposition, which followed from their TGA thermograms (Figure 3). The process was conducted for 4 h. During this time, samples containing 15 masses. % m- and p-AAP were completely solidified. Specimens containing o-AAP did not turn away even for a longer time.

The soluble fraction was extracted from all warmed-up samples using the Soxlet device. For this, 1,4-dioxane was used as a solvent as it is capable of dissolving both DER-331 and all of the AAP. As can be seen in Table 1, in the case of m- and p-AAP, the gel fraction content in the samples of the cured compositions is increasing, indicating a step-by-step mechanism of resin curing. In addition, at 15 mass. % m- and p-AAP in the composition, the gel fraction content reaches 97% and 98%, respectively, which confirms that the AAP chemically bind to epoxy resin to form a 3D polymer matrix.

As for the o-AAP, such a low gel fraction is most likely due to steric difficulties. B-AAP, in addition to the bulky benzene and phosphazene rings, limits the access to the -NH- group to the closely spaced methyl group. This assumption is consistent with the studies in reference [23], which provide calculations of the structural parameters of o-methylaniline both in the basic and in the excited singlet states at the levels of the theory MP2/6-31+G* and CIS/6-31+G*; they also show the influence of the methyl group on the amino group and prove the significant contribution of the steric factor to the availability of the nitrogen atom.

**Figure 8:**
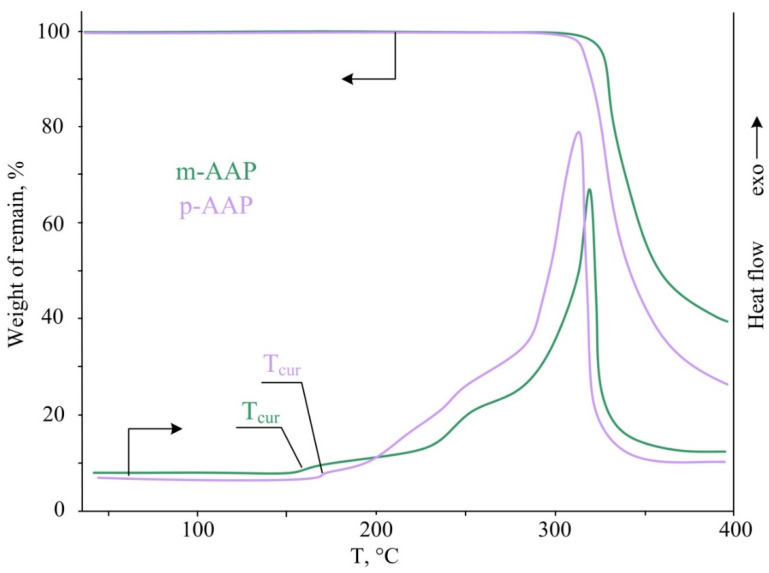
TGA and DSC thermogram of compositions based on DER-331 containing 15 mass. % m- or p-AAP (arrows show which scale corresponds to which thermograms).

To optimize the curing conditions, compositions containing 15% m-AAP and p-AAP have been solidified under the synchronous TGA-DSK experiment. The thermograms produced by DSK (Figure 8) show that the resin solidification begins at 160 °C in the case of m-AAP and 160 °C in the case of p-AAP. This means that in order to produce a homogeneous polymer, the composition must be heated quickly because, as previously stated, the AAP are only completely dissolved at 200 °C and above. If the solidification is slow, a mesh polymer will form on the AAP surface and no further AAP dissolution will occur in the resin.

Additionally, from the DSC it follows that with the increase in the curing temperature, the thermal effect increases, which indicates an increase in the speed of the process. At the same time, at the melting point m- and p-AAP (Figure 3), when the resin solidifies, the melting peaks are no longer observed, which indicates that the crystalline structure of the phosphazenes is being destroyed by the resin. The TGA curves show that the hardening process does not cause the m- and p-AAP thermal degradation, which started at clean compounds at 250 °C. This is due to the fact that during the solidification process, a proton migrates from the nitrogen -NH- to the oxyrane cycle, instead of forming the C-N bond (Figure 6). Therefore, the formation of aniline becomes impossible. The solidification process for both of the AAP is accelerated up to 300 °C to form a thermostable polymer, after which the degradation process begins, accompanied by rapid mass loss. Therefore, compositions based on DER-331 and m-, p-AAP can be used for the preparation of products in the range of 200–300 °C. In the DSC analysis of solidified compositions, their glass transition temperatures were determined, which were 80 and 85 in the case of the use of m-AAP and p-AAP, respectively.

The polymers produced on the basis of DER-331 and m-, p-AAP have been tested for fire resistance according to UL-94. For horizontal fixing, the fire resistance of the consumption category HB is for the vertical attachment of the fire-resistance category V-0, i.e., the polymers are non-combustible.

## 4. Conclusions

Since the developed compositions based on the industrial epoxy resin DER-331 and arilaminophosphazenes m- and p-AAP arilaminocyclotriphosphazenes can be converted into products at 200–300 °C, the best use for them is as a press binderpowders and as hot curing adhesives. The glass transition temperatures of the produced polymers are 80 and 85 °C, which are quite acceptable for use in domestic conditions. In addition, the polymers are non-combustible, which allows them to operate products in facilities with a high fire hazard.

## Figures and Tables

**Table 1:** Dependence of gel fraction content in cured epoxy compositions depending on the content of modifier therein.

Curing Agent	Temperature, °C	Mass Loss, %
M-AAP	5	1.77
	10	11.65
	15	97.07
P-AAP	5	4.48
	10	43.93
	15	90.36
O-AAP	5	0
	10	2.76
	15	2.94

## Data Availability

The data presented in this study are available on request from the corresponding author.
